# Retrospective observation of subacute thyroiditis before and during the COVID-19 vaccination campaign in a single secondary endocrine centre in the Savona district, Liguria, Italy

**DOI:** 10.1186/s13044-022-00139-z

**Published:** 2022-11-02

**Authors:** Massimo Giusti, Marilena Sidoti

**Affiliations:** 1 Endocrine Unit, Priamar Clinical-Diagnostic Centre, Savona, Italy; 2 Local Health Service 2 Savonese, Liguria Region, Sidoti Italy; 3 Centro Clinico-Diagnostico Primar, via dei Partigiani 13R, I-17100 Savona, Italy

**Keywords:** COVID-19, Subacute thyroiditis, Outcome

## Abstract

**Background:**

Clinicians should be aware that subacute thyroiditis (SAT) might be an under-reported adverse effect of COVID-19 vaccines.

**Aim:**

In records from endocrinological examinations, we reviewed the incidence of diagnoses of SAT from 2000 to 2020 and during the 2021 COVID-19 vaccination campaign.

**Methods:**

Review of electronic records from June to December in each year from 2000 to 2021.

**Results:**

From 2000 to 2020, 51 patients in our centre had SAT (0.6%). From June to December 2021, 7 females were diagnosed with SAT after vaccination. The percentage of SAT in 2021 medical files was 1.5%. SAT diagnoses significantly (P = 0.03) increased in 2021 in comparison with the 2000–2020 period. The median age of SAT patients in 2021 (51 years; IQR 35–66 years) was higher than in the 2000–2020 period (45 years, IQR 38–52 years; P = 0.05).

**Conclusion:**

To date, few cases of SAT after COVID-19 vaccinations have been described in the literature, with sub-clinical, normal or increased thyroid function during 1-3-month follow-up. Our findings indicate that SAT after COVID-19 vaccination occurs more frequently than in other virus-related cases and at a greater age. Our observation of a local increase in SAT during the 2021 COVID-19 vaccination campaign indicates that physicians should be aware of this infrequent side effect, which must be considered and monitored after COVID-19 vaccination.

## Background

Subacute thyroiditis (SAT) due to viral infections is an infrequent cause of thyrotoxicosis [1]. Common symptoms are neck pain and tenderness on palpation [1].

In the COVID-19 era, SAT seems to be an underestimated consequence of the disease which occurs during SARS-CoV-2 infection [2]. In 2021, some cases were also reported after the administration of COVID-19 vaccines [3]. We reviewed the incidence of SAT from 2000 to 2021 in a private secondary endocrine centre in the Savona district of Liguria (Italy), which has a population of about 280,000. Vaccination of the general population started in February 2021 and progressively involved fragile, healthy elderly, adult and young-adult subjects. By the end of 2021, about 80% of adults in the Savona district had received at least two doses of COVID-19 vaccines.

The aim of this study was to compare the incidence of SAT in these two periods: before and during the 2021 COVID-19 vaccination campaign.

## Methods

The study was conducted in the private secondary Priamar Centre for out-patients. Electronic records of all endocrinological examinations from June to December in each year from 2000 to 2020 were searched. From 8115 medical files, 51 diagnoses of SAT were anonymously retrieved (35 females; 16 males). In the corresponding period in 2021, in 466 medical files, SAT was diagnosed in 7 women after COVID-19 vaccination. The diagnosis was made according to the following findings: neck pain and tenderness on palpation, focal or diffuse hypoechogenicity and/or heterogeneous echostructure of the thyroid on ultrasonography (US), increased inflammatory makers [erythrocytesedimentation rate (ESR) and/or C-reactive protein (CRP)], and transient clinical signs of mild/moderate thyrotoxicosis. After the onset of illness, thyroid function tests [free-thyroxine(f-T4), thyroid stimulating hormone (TSH)] and thyroid autoimmunity [thyroperoxidaseantibodies (TPOAb); TSH-receptor antibodies(TRAb)] were carried out by means of commercial assays in the laboratory of Local Health Service 2 Savonese. Analytic normal ranges are: ESR < 25 mm/h, CPR < 5 mg/l, f-T4 12.0–22.0 pmol/l, TSH 0.3–4.2 mIU/l, TRAb < 0.7 mIU/l and TPOAb (negative according to the manufacturers). Non-parametric tests (Mann-Whitney test, Fisher’s exact test) were used. The study was approved by the Priamar Centre’s institutional board, and a waiver of informed consent was granted because the research involved no risk to patients.

## Results

From 2000 to 2020, about 0.6% of the patients evaluated in our centre had SAT, which occurred at the median age of 45 years (IQR 38–52 years) with an incidence of 1–3/year. The long-term evolution of the disease was known in 25 cases (49%) and full thyroid recovery or chronic thyroiditis/hypothyroidism was observed in 72% and 28% of cases, respectively. From June to December 2021, 7 women were diagnosed with SAT after vaccination (Table 1). The percentage of SAT in 2021 medical files was 1.5%. SAT diagnoses significantly (P = 0.03) increased in 2021 in comparison with the 2000–2020 period. The median age of SAT patients in 2021 (51 years; 35–66 years) was significantly higher than in the 2000–2020 period (P = 0.05). To date, the median follow-up has been 12 weeks (8–29 weeks). In 3 women, a decrease in thyroid volume was noted. When values were available (n = 5) on first examination, TSH was suppressed in 80% of patients. At the last examination, TSH was still suppressed in 2 and had increased in 2; in the remaining patient, L-T4 was still ongoing. Pain, palpitations, fatigue and sweating did not recur after discontinuation of prednisone/FANs (Table 1).

**Table 1:** Clinical and laboratory data collected after COVID-19 vaccination in patients with diagnosis of SAT

Case	Age years	Vaccine type (#)	Weeks after vaccination	Symptoms	f-T4 pmol/l	TSH mIU/l	TPOAb P/N	TRAb mIU/l	CRP mg/l	US findings (*)	Therapy
1	35	mRNA-1273, 2^nd^	2	neck pain	12.6	< 0.03	nd	nd	nd	nd	FANs
			6	palpitation	7.6	< 0.03	P	0.5	102	diffuse, 5	none
			14	none	10.6	0.11	P	nd	12	focal, 6	none
2	42	mRNA-1273, 1^st^	3	neck pain	26.1	0.01	P	nd	nd	diffuse, nd	FANs
			8	none	nd	4.46	nd	< 0.3	25	normal, 8	none
3	50	BNT162b2, 2^nd^	6	neck pain	16.0	0.06	nd	nd	nd	diffuse, nd	FANs
			10	palpitation	20.6	3.03	N	< 0.3	48	normal, 12	none
4	51	ChAdOx1, 1st	4	neck pain	nd	nd	nd	nd	10	diffuse, 20	none
			9	weight gain	5.8	10.4	N	< 0.3	4	diffuse, 5	none
			29	none	12.0	5.30	N	nd	nd	diffuse, 4	L-T4
5	54	BNT162b2, 2^nd^	3	neck pain	18.5	0.69	N	nd	nd	diffuse, 13	prednisone
			10	none	11.3	7.60	nd	nd	33	normal, 5	none
6	61	mRNA-1273, 1^st^	4	palpitation	15.6	< 0.03	N	nd	nd	focal, 9	B-blocker
			13	none	7.9	0.09	N	0.4	18	normal, 8	B-blocker
7	63	BNT162b2, 1^st^	4	neck pain	nd	nd	nd	nd	44	diffuse, 18	prednisone
			12	weight gain	12.4	2.20	N	nd	46	focal, 10	B-blocker

Legend: nd, not done; #, 1^st^ or 2^nd^ vaccine administration before onset of SAT; P/N, positive/negative TPOAb titer; US diffuse, focal hypoechoic pattern; (*) the number indicates thyroid volume in milliliters; FANs, non-steroidal anti-inflammatory drugs

## Discussion

In an old study conducted in the state of Minnesota, USA, during the period 1960–1997, the incidence of SAT was reported as 4.9 cases per 100,000/year [4]. The Priamar Centre performs only some of the endocrine examinations in our district, where other primary public and private centres also operate; this might explain the raw 3-fold lower number of SAT cases observed. However, in 2021, when the COVID-19 vaccination campaign was at its height, we observed a significant increase in SAT. Here, we describe SAT cases that emerged 2–6 weeks (median 4 weeks) after several types of vaccines against the SARS-CoV-2 virus. To date, several cases of SAT after COVID-19 vaccinations have been described in the literature, with sub-clinical, normal or increased thyroid function in about 29%, 53% and 12% of cases, respectively, during 1-3-month follow-up (see ref 3). Recently, Bahçecioğlu et al. [5] reported a 9.3% incidence of SAT after SARS-Cov-2 vaccination between March 2020 and July 2021, and compared this percentage with that recorded from March 2018 to July 2019. The authors did not observe a significant increase in the total number of SAT cases during the pandemic [5]. However, there are several differences between their study and ours, both in the setting (length of the reference period, inclusion in the Turkish study of both patients with SAT due to COVID-19 disease and those with SAT due to SARS-CoV-2 vaccination, secondary vs. tertiary care) and in the population analysed, which might display different HLA-based genetic susceptibility, as recently reported [6]. These differences make it difficult to compare the two studies.

SAT affects women almost 4 to 5 times as often as men and typically occurs between 25 and 35 years of age (1), although higher ages on SAT presentation have been reported [7, 8]. SAT after SARS-CoV-2 vaccines seems preferentially to involve women [3], and indeed, all 7 cases observed by us in 2021 involved women. It is well known that the incidence of SAT decreases with increasing age [1]; however, we observed an increase in the age of SAT patients after 2021 vaccination in comparison with cases diagnosed in the period 2000–2020. We can speculate that this adverse event increases in parallel with the higher risk of severe COVID-19 disease complications in middle-aged and elderly subjects than in young adults. Autoimmunity is not significant in SAT [1] and TPOAb positive titres were found in only 2 of our patients in 2021. Like other viral infections [1], SARS-CoV-2 is a cause of SAT [2], and SARS-CoV-2 viral proteins have recently been detected in the thyroid tissue together with histopathological characteristics of SAT [9]. SAT after COVID-19 vaccination seems to be due to some structural similarities between follicular thyroid cells and SARS-CoV-2 antigens. In addition, the adjuvants used to induce a more substantial and sustained humoral and cellular immune response can trigger adverse immune reactions in predisposed individuals, causing autoimmune/inflammatory syndrome [10].

In conclusion, our observation of a local increase in SAT during the 2021 COVID-19 vaccination campaign indicates that physicians should be aware of this infrequent side effect, which must be considered and monitored after COVID-19 vaccination.

## Data Availability

The datasets used and/or analysed in the present study are available from the corresponding author on reasonable request.

## List of abbreviations

SAT: Subacute thyroiditis.
COVID-19: Coronavirus disease.
IQR: interquartile range.
SARS-CoV-2: severe acute respiratory syndrome coronavirus 2.
US: Ultrasonography.
CRP: C-reactive protein.
TSH: Thyroid stimulating hormone.
f-T4: Free-thyroxine.
TPOAb: Thyroperoxidase antibodies.
TRAb: TSH-receptor antibodies.
